# Severe *Candida glabrata* pancolitis and fatal *Aspergillus fumigatus* pulmonary infection in the setting of bone marrow aplasia after CD19-directed CAR T-cell therapy – a case report

**DOI:** 10.1186/s12879-020-05755-4

**Published:** 2021-01-28

**Authors:** Kai Rejeski, Wolfgang G. Kunz, Martina Rudelius, Veit Bücklein, Viktoria Blumenberg, Christian Schmidt, Philipp Karschnia, Florian Schöberl, Konstantin Dimitriadis, Louisa von Baumgarten, Joachim Stemmler, Oliver Weigert, Martin Dreyling, Michael von Bergwelt-Baildon, Marion Subklewe

**Affiliations:** 1 Department of Hematology and Oncology, University Hospital, LMU Munich, Munich, Germany; 2 Laboratory for Translational Cancer Immunology, LMU Gene Center, Munich, Germany; 3 German Cancer Consortium (DKTK) and German Cancer Research Center, Heidelberg, Germany; 4 Department of Radiology, University Hospital, LMU Munich, Munich, Germany; 5 Department of Pathology, University Hospital, LMU Munich, Munich, Germany; 6 Department of Neurosurgery, University Hospital, LMU Munich, Munich, Germany; 7 Department of Neurology, University Hospital, LMU Munich, Munich, Germany

**Keywords:** CAR T-cell, Case report, Hematotoxicity, *Candida glabrata*, Invasive aspergillosis

## Abstract

**Background:**

Prolonged myelosuppression following CD19-directed CAR T-cell transfusion represents an important, yet underreported, adverse event. The resulting neutropenia and multifactorial immunosuppression can facilitate severe infectious complications.

**Case presentation:**

We describe the clinical course of a 59-year-old patient with relapsed/refractory DLBCL who received Axicabtagene-Ciloleucel (Axi-cel). The patient developed ASTCT grade I CRS and grade IV ICANS, necessitating admission to the neurological ICU and prolonged application of high-dose corticosteroids and other immunosuppressive agents. Importantly, neutropenia was profound (ANC < 100/μl), G-CSF-refractory, and prolonged, lasting more than 50 days. The patient developed severe septic shock 3 weeks after CAR transfusion while receiving anti-fungal prophylaxis with micafungin. His clinical status stabilized with broad anti-infective treatment and intensive supportive measures. An autologous stem cell backup was employed on day 46 to support hematopoietic recovery. Although the counts of the patient eventually started to recover, he developed an invasive pulmonary aspergillosis, which ultimately lead to respiratory failure and death. Postmortem examination revealed signs of *Candida glabrata* pancolitis.

**Conclusions:**

This case highlights the increased risk for fatal infectious complications in patients who present with profound and prolonged cytopenia after CAR T-cell therapy. We describe a rare case of *C. glabrata* pancolitis associated with multifactorial immunosuppression. Although our patient succumbed to a fatal fungal infection, autologous stem cell boost was able to spur hematopoiesis and may represent an important therapeutic strategy for DLBCL patients with CAR T-cell associated bone marrow aplasia who have underwent prior stem cell harvest.

## Background

When CD19-directed Chimeric Antigen Receptor (CAR) T-cell therapies were first introduced into the clinical setting, a set of distinctive side effects were observed, including Cytokine Release Syndrome (CRS) and immune effector cell-associated neurotoxicity syndrome (ICANS). As these treatments transition from large clinical trials to real-world implementation, new nuances of the side effect spectrum have emerged. This includes the observation that patients can present not only with grade 3–4 cytopenia (CTCAE v5.0), but also a syndrome of persistent cytopenia after CAR T-cell transfusion [1, 2]. Hematologic toxicity is common with one study demonstrating that neutropenia, thrombocytopenia and anemia occur in 94, 80 and 51% of patients respectively [3]. Risk factors for hematotoxicity include CRS grade, baseline cytopenia, and prior allogeneic stem cell transplantation within the last year [2, 3]. The fact that baseline hematopoietic reserve contributes to cytopenia in CAR T-cell patients may explain the variability in the incidence of hematoxicity in the real-world setting compared to the large registration trials, which set stringent hematologic exclusion criteria [4, 5]. The clinical utility of therapeutic rescue strategies that mitigate the risk of infectious complications secondary to long-term bone marrow (BM) aplasia, such as early autologous stem cell boost or Eltrombopag, remains unknown. Moreover, the relative paucity of reports concerning severe and life-threatening infections in the reported CD19-directed CAR T-cell trials necessitates the presentation of distinctive clinical courses that underline the risks of persistent cytopenia after CAR T-cell therapy in a real-world setting.

## Case presentation

**Fig. 1:**
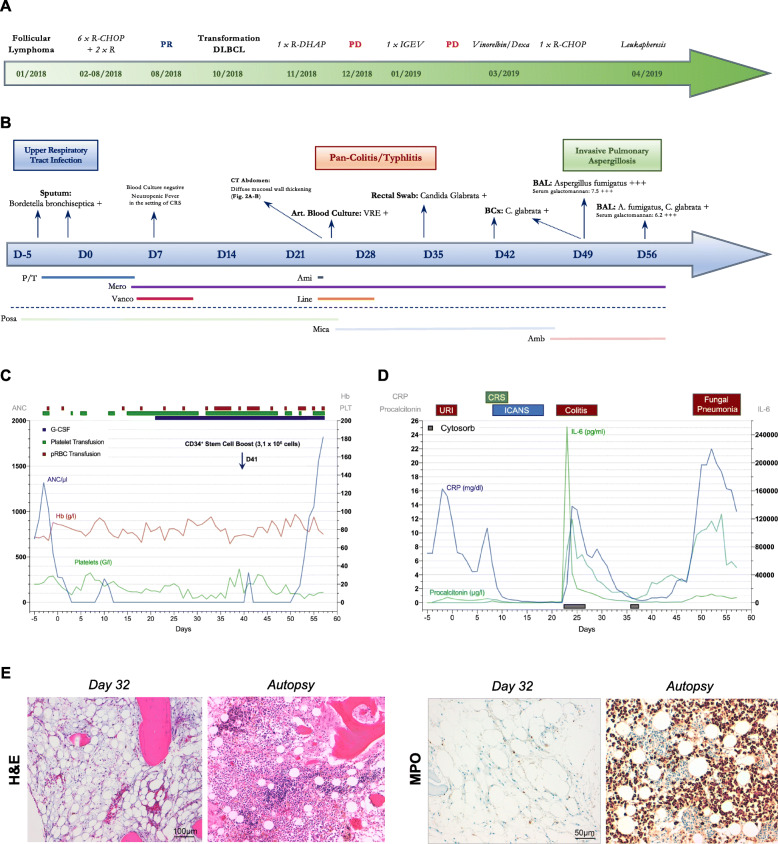
Overview of CAR T-cell mediated hematotoxicity and infectious complications. **a** Treatment course prior to CAR T-cell therapy. **b** Timeline of infectious complications during CAR T-cell treatment course. Positive microbiologic culture results are underlined. Bottom: Overview of utilized anti-infectives with the respective bars displaying the length of treatment. P/T = piperacillin/tazobactam, Ami = amikacin, Mero = meropenem, Vanco = vancomycin, Line = linezolid, Posa = posaconazole (p.o.), Mica = micafungin (i.v.), Amb = liposomal amphotericin B. The patient also received prophylactic acyclovir and sulfamethoxazole/trimethoprim (TMP/SMZ) during treatment. **c** Complete Blood Count (CBC) timeline. ANC (blue), platelet count (green), Hemoglobin (red). Transfusion events (green: platelet transfusion, red: pRBC transfusion) and G-CSF support (blue bar) are integrated in the curve. **d** Dynamics of Serum Inflammatory Markers. Infectious complications are superimposed above the graph. **e** Histopathologic analysis of BM biopsies demonstrating severe BM aplasia 1 month after CAR T-cell transfusion (Day 32, upper panel) and evidence of recovering hematopoiesis following autologous stem cell transfer (Autopsy, lower panel). Immunohistochemical staining for myeloperoxidase (= MPO) highlighting strong activation of myelopoiesis (right panel)

**Fig. 2:**
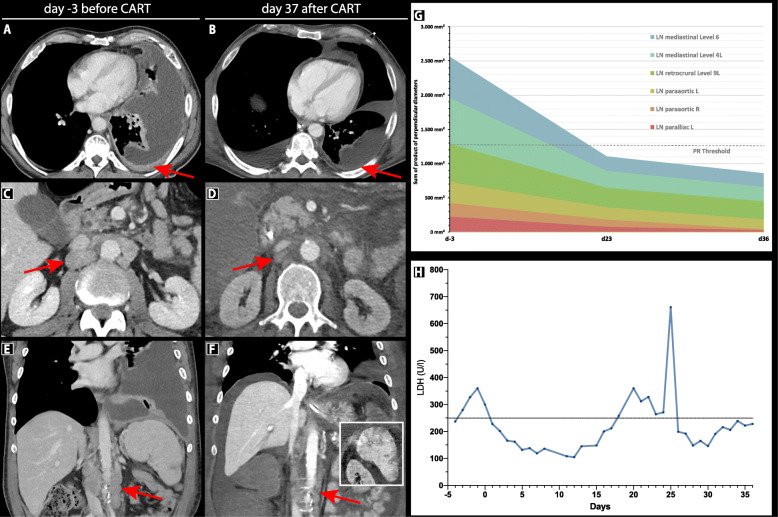
Radiographic evidence of partial response to Axi-cel after 36 days. Contrast-enhanced CT abdomen (axial view) displaying pleural manifestations before (**a**) and after (**b**) CAR T-cell treatment. Involved paraaortic lymph nodes (red arrows) demonstrate an interval decrease in size (**c-d**, **e-f**). The inset of panel F depicts splenic hypodensities consistent with necrosis of tumor tissue secondary to immune therapy and a reduction of splenomegaly. **G** Graphical depiction of response over time. Tumor extent was quantified as the sum of the product of perpendicular diameters according to Lugano criteria over 3 time points and is graphed on the y-axis. **H** Timeline of LDH levels (U/l). The increase in LDH levels at day 25 correlated with the gastrointestinal toxicity and liver dysfunction

**Fig. 3:**
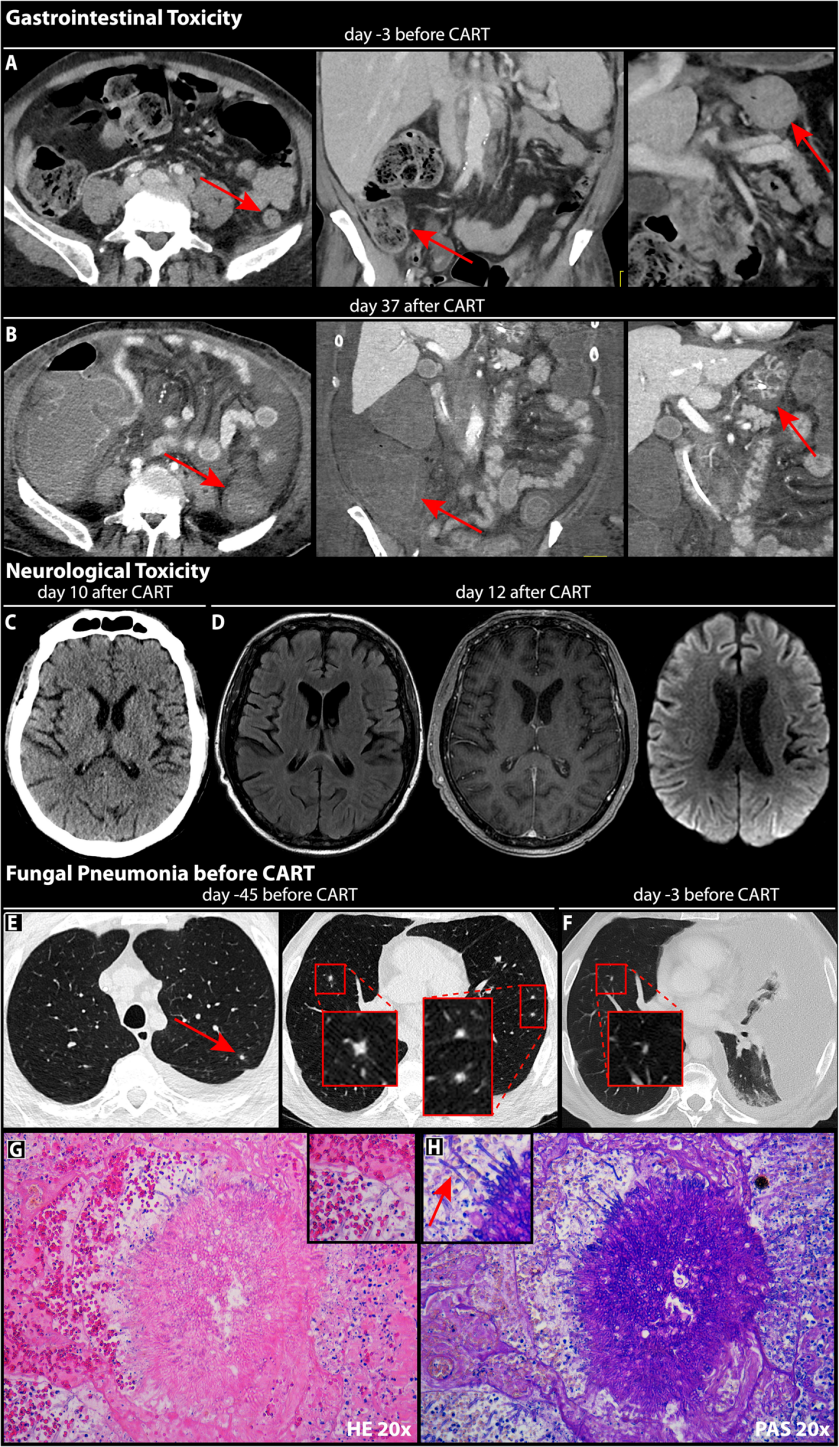
Multi-organ toxicity secondary to CD19-directed CAR T-cell therapy. **a** Contrast-enhanced CT abdomen demonstrating a non-inflamed baseline state of GI mucosal tissue prior to CAR T therapy. **b** Panmural swelling of the sigma (left panel), ascending colon (middle panel), and stomach. **c** Non-pathologic CT of the brain. **d** Axial FLAIR-weighted (left), T1-weighted contrast-enhanced (middle), and diffusion-weighted (right) MRI displaying no evidence of cerebral edema, white matter lesions, diffusion restriction, microbleeds, meningeal enhancement or contrast enhancing lesions. **e** High-resolution CT thorax with bilateral fungal infiltrates observing the typical halo configuration 6 weeks prior to CAR T-cell transfusion. **f** On day − 3 the right-sided infiltrates have decreased in size. The left lung is collapsed due to malignant pleural effusion. **g** Lung parenchyma exhibiting an infiltration of non-dimorphic blastoconidial morphology consistent with *Candida glabrata*. **h** PAS staining of a lung specimen highlights septated hiphae and acute-angle branching, features consistent of with aspergillus infection

The 59-year-old male patient was initially diagnosed with stage IIIA follicular lymphoma in January 2018, which transformed to germinal center B-cell like DLBCL in October of the same year. Refractory to multiple cycles of Rituximab-based immunochemotherapy (Fig. 1a), the patient underwent leukapheresis for CAR T-cell therapy in April 2019. The patient experienced significant lymphoma progression during the manufacturing process of Axicabtagene ciloleucel (Axi-cel), manifesting itself in the form of malignant pleural effusions and progressive lymphadenopathy (Fig. 2a,c,e). In the six-week period between leukapheresis and lymphodepletion, the patient remained severely neutropenic after R-CHOP and developed possible invasive fungal disease (IFD) [6] (Fig. 3e) as well as *E. coli* septicemia secondary to a urinary tract infection. These infections were treated successfully with liposomal amphotericin B and piperacillin/tazobactam. Prior to lymphodepletion (D-5), the patient presented with an ECOG performance status of 1, intact renal and liver function, while observing residually depressed blood counts (WBC 1270/μl, ANC 700/μl, ALC 390/μl, Hemoglobin 7.2 g/dl, Platelets 20 G/l). BM histopathology showed a hypocellular marrow with no evidence of lymphoma infiltration. Analysis of lymphocyte subpopulations demonstrated severe B-cell aplasia and decreased absolute CD4+ (84/μl) and CD8+ (271/μl) counts. His anti-infective prophylaxis consisted of a combination of acyclovir, sulfamethoxazole/trimethoprim (TMP/SMZ), and posaconazole. The fludarabine/cyclophosphamide lympho-preparative regimen was applied on days − 5 to − 3 according to the provider’s protocol, followed by CAR T-cell transfusion on day 0.

During lymphodepletion the patient developed an upper respiratory infection, presenting with a non-productive cough and a sputum culture positive for *Bordetella bronchiseptica*, which resolved clinically after antibiotic treatment with piperacillin/tazobactam. On day 7, the patient developed fever with a stable cardiopulmonary function and concomitant IL-6 rise (Fig. 1d), which was classified as CRS grade I according to ASTCT consensus grading [7] in the absence of positive blood cultures and other foci. Due to persistent fever refractory to antipyretic treatment, four total doses of tocilizumab (8 mg/kg) were applied over 36 h. Starting on day 9, the patient deteriorated neurologically with a depressed state of consciousness prompting the diagnosis of grade IV ICANS. The patient was transferred to the neurological ICU, and high-dose corticosteroids (methylprednisolone 1000 mg daily) were initiated. CSF-analysis displayed subtle pleocytosis, but revealed no signs of CNS infection (viral, bacterial and fungal) or meningeal involvement of the DLBCL. CT and MR imaging of the brain exhibited none of the pathological findings reported for ICANS [8] or CNS infection [9](Fig. 3c-d). After 5 days of high-dose methylprednisolone, the patient’s neurological status slowly improved, and corticosteroids could be tapered.

### Partial response to Axicabtagene-Ciloleucel after 36 days according to Lugano criteria

While the patient’s initial clinical course was fraught with complications, CT staging performed at day 36 demonstrated a partial response to CAR T-cell therapy according to Lugano and Lyric criteria (Fig. 2a-g). This was particularly evident for the pleural manifestations of the underlying DLBCL, which was accompanied by an interval decrease in pleural effusions and subjective dyspnea (Fig. 2a-b). Multiple nodal sites including paraaortic (Fig. 2c-d) and parailiac lymph nodes (Fig. 2e-f) exhibited significant regression of tumor size (Fig. 2g). Moreover, the patient displayed splenic hypodensities consistent with either tumor tissue necrosis or infection-associated inflammatory changes (inset, Fig. 2f). These CT-morphologic findings were accompanied by a decrease of LDH levels (Fig. 2h).

### Prolonged cytopenia lead to the development of severe neutropenic colitis and fatal fungal pneumonia

The patient presented with significant myelotoxicity and sustained transfusion dependency after CAR T-cell administration (Fig. 1c). The overall duration of profound neutropenia – defined as an ANC < 100/μl [10] – was 52 days. Because of the severity of ICANS, the clinicians chose to hold G-CSF during this time period due to concerns for risk of worsening toxicity [11].

While receiving broad anti-infectives (acyclovir, TMP/SMZ, posaconazole, meropenem), the patient developed breakthrough septic shock on day 24. He exhibited fever (39.5 °C), hypotension (70/30 mmHg), tachycardia (160/min), as well as lactic acidosis (lactate: 10.7 mmol/l). The patient was non-conversant, non-oriented, and was not able to localize symptoms. However, he had described epigastric tenderness and subtle changes in stool consistency and frequency on the previous day. As a result, abdominal CT imaging was obtained, which displayed severe pancolitis, as evidenced by pronounced mucosal thickening with edematous changes ranging from the ascending colon to the rectum (Fig. 3a-b). Labs were notable for an acute spike of serum inflammatory parameters (Il-6 > 500.000 pg/ml, CRP 5.7 mg/dl, Procalcitonin 11.2 ng/ml, Fig. 1d). Following ICU transfer, high-dose vasopressor support with norepinephrine was initiated together with volume resuscitation to maintain organ perfusion. Due to progressive loss of consciousness the patient was intubated and mechanically ventilated. To control the proinflammatory state, extracorporeal cytokine absorption (Cytosorb®) was performed, which resulted in a decrease of soluble inflammatory markers in the subsequent days. Arterial blood cultures were positive for vancomycin-resistant enterococci (VRE), and the antibiotic treatment was escalated to include linezolid along with meropenem (Fig. 1b). The ICU team transitioned antifungal prophylaxis from posaconazole to an echinocandine (micafungin 50 mg IV) due to ECG changes and concerns for drug interactions on day 24. Though he remained in critical condition, in the coming weeks his clinical condition stabilized and both the catecholamine rate and volume support could be reduced. To determine the origin of sustained myelosuppression, a bone marrow biopsy was obtained, which depicted a hypocellular marrow and severe aplasia affecting all hematopoietic lineages (Fig. 1e). On days 41 and 49, surveillance blood cultures were positive for *Candida glabrata*, a commensal fungal organism of human mucosal tissues [12] harboring an intrinsic azole resistance [13]. Susceptibility testing confirmed the azole-resistance of *C. glabrata* isolates. The antimycotic therapy was escalated from micafungin to liposomal amphotericin B. *C. glabrata* likely disseminated to the bloodstream secondary to the described gastrointestinal toxicity and was previously discovered on rectal swab (Fig. 1b) and fecal culture (day − 51).

Due to sustained neutropenia, BM aplasia and the availability of a suitable apheresis product, the CAR T-cell Taskforce proceeded with a stem cell boost of previously collected autologous CD34+ cells (3,1 × 10^6^ cells) on day 46. The combination of a sudden rise in serum inflammatory markers and a strongly positive serum galactomannan (GM) assay on day 49 prompted diagnostic bronchoscopy, which revealed a diffuse infiltrate of alveolar and peri-bronchial tissue by *Aspergillus fumigatus*, consistent with fulminant invasive fungal pneumonia. Over the next week, the patient became increasingly hemodynamically unstable with poor peripheral oxygenation. Repeat bronchoscopy with bronchial lavage was positive for *A. fumigatus* and *C. glabrata*. After 50 days of G-CSF refractory aplasia, the autologous stem cells slowly engrafted leading to neutrophil recovery. However, this likely exacerbated the local inflammatory reaction in the lung, contributing to rapid deterioration of his respiratory status. Despite extensive supportive measures, the patient died on day 58 after CAR T-cell transfusion. Cause of death was confirmed by autopsy as invasive fungal pneumonia with multi-organ septic spread. Post-mortem examination revealed Candida spores in intestinal, colonic and rectal tissue. Of note, histopathologic BM analysis revealed signs of burgeoning hematopoietic regeneration (Fig. 1e).

## Discussion and conclusions

Recent studies have shed light on the high incidence of prolonged and profound neutropenia after adoptive immunotherapy with CD19-specific CAR T-cells [1–3]. Clinical sequalae include severe infectious complications, which have emerged as the number one cause of long-term non-relapse mortality after CAR T-cell therapy [14]. In a seminal study, Hill and colleagues demonstrate that infectious complications are common with a cumulative incidence of 23% in the first 28 days after CAR T-cell transfusion and 14% between days 29–90. While bacterial infections were most common in the first month, viral infections – especially respiratory viruses – predominated after day 29, likely as a result of an impaired adaptive immune response due to B-cell aplasia [15]. Overall, most infections were mild to moderate and fatal infections were rare. Invasive mold infections were uncommon, ranging from 1 to 7% in CAR T-cell recipients [15–17]. In multivariate analyses, the use of systemic corticosteroids for the management of CRS or ICANS has emerged as a major risk factor of infection [18]. Certain inflammatory signatures – such as the “double peaks of IL-6” pattern observed in our patient – have been shown to confer an especially high risk of life-threatening infection [19].

This case highlights the combination of factors that precipitate a high risk of infectious complications in a CAR T-cell patient. First, the heavily pre-treated patient displayed B-cell aplasia and a diminished hematopoietic reserve (e.g. trilineage cytopenia) prior to lymphodepletion. Second, the patient developed classic complications of CAR T-cell therapy such as CRS and severe and prolonged ICANS, reflecting a pro-inflammatory state that represents an important risk factor for developing infections in CAR T-cell patients [16]. The subsequent application of high-dose corticosteroids impaired both innate and adaptive immunity [20]. Third, pancytopenia was profound, prolonged and not able to be reverted by growth factor support. Fourth, the patient had a history of infectious complications, including a course of fungal pneumonia 6 weeks prior to lymphodepletion, likely flaring up in the setting of the above three factors.

Our patient developed severe septic shock secondary to neutropenic colitis on day 24, highlighting the risk of a watch-and-wait approach to prolonged neutropenia after CAR T-cell therapy. A two-pronged approach to management appears prescient. On the one hand, measures to propagate hematopoietic recovery should be exhausted (causal therapy). This can range from *G-CSF* stimulation, to a trial of pulse-dose corticosteroids or anti-cytokine therapy (e.g. Anakinra, Tocilizumab), to a stem cell boost as a last resort in lymphoma patients who have undergone prior autologous stem cell transplantation. On the other hand, clinicians should be at high alert for infectious complications and adapt their infection surveillance and anti-infective prophylactic strategies accordingly. For example, therapeutic drug monitoring of posaconazole and meropenem levels may have revealed subtherapeutic dosing of these anti-infective agents in this patient [21]. The role of antifungal prophylaxis in the CAR T-cell patient remains inconclusive. Adequately powered studies addressing the duration of neutropenia after CAR T-cell therapy, and potential clinical determinants of invasive fungal infections, will be critical to gauge the benefit of antifungal prophylaxis in this patient collective.

Candida species are a leading cause of fungus-associated morbidity and mortality in severely immunocompromised patients [21]. On day 41, this patient developed breakthrough *C. glabrata* Candidemia on micafungin, in the setting of both underlying colonization and neutropenic colitis. A recent report implicated the gastrointestinal tract as major source of echinocandin drug resistance in a murine model of *C. glabrata* colonization and systemic dissemination [22]. Of note, breakthrough Candida infections on micafungin may occur at the employed dose level (50 mg/day IV), suggesting that this dose may have been inadequate to prevent subsequent Candidemia [23]. Though cases of *C. glabrata* colitis are extremely rare [24], an opportunistic infection could not be ruled out given that *C. glabrata* infiltrates were discovered in enteral and colonic mucosal tissue on post-mortem examination. Importantly, the patients’ clinical status deteriorated only after the stem cell boost resulted in signs of hematopoietic recovery. The paradoxical worsening of the pulmonary *A. fumigatus* infection is consistent with immune reconstitution inflammatory syndrome (IRIS), which has been previously described for HIV/AIDS [25] and for severely neutropenic patients with IPA [26]. In conclusion, this case emphasizes that infectious complications secondary to prolonged cytopenia influence non-relapse mortality after CAR T-cell therapy.

## Data Availability

The authors did not use any database, software, or tools for the writing of this manuscript.

## Abbreviations

ALC: Absolute Lymphocyte Count
ANC: Absolute Neutrophil Count
BM: Bone Marrow
CAR: Chimeric antigen receptor
CBC: Complete Blood Count
CRS: Cytokine release syndrome
CTCAE: Common Terminology Criteria for Adverse Events
DLBCL: Diffuse large B-cell lymphoma
FFP: Fresh frozen plasma
FLAIR: Fluid-attenuated inversion recovery
Flu/Cy: Fludarabine/Cyclophosphamide
G-CSF: Granulocyte colony stimulating factor
GI: Gastrointestinal
GM: Galactomannan
HSPCs: Hematopoietic stem and progenitor cells
ICANS: Immune effector cell-associated neurotoxicity syndrome
ICE-score: Immune Effector Cell-associated Encephalopathy score
ICU: Intensive care unit
IPA: Invasive pulmonary aspergillosis
LDH: Lactate Dehydrogenase
LYRIC: Lymphoma response to immunomodulatory therapy criteria
MDRD: Modification of Diet in Renal Disease
MPO: Myeloperoxidase
PAS: Periodic acid-Schiff
PBSC: Peripheral Blood Stem Cell
VRE: Vancomycin-resistant Enterococcus
WBC: White Blood Count
